# First nationwide survey on cardiovascular risk factors in Grand-Duchy of Luxembourg (ORISCAV-LUX)

**DOI:** 10.1186/1471-2458-10-468

**Published:** 2010-08-10

**Authors:** Ala'a Alkerwi, Nicolas Sauvageot, Anne-Françoise Donneau, Marie-Lise Lair, Sophie Couffignal, Jean Beissel, Charles Delagardelle, Yolande Wagener, Adelin Albert, Michèle Guillaume

**Affiliations:** 1 Centre de Recherche Public Santé, Centre d'Etudes en Santé, Grand-Duchy of Luxembourg; 2 School of Public Health, University of Liège, Belgium; 3 Centre Hospitalier du Luxembourg, Grand-Duchy of Luxembourg; 4 Directorate of Health, Ministry of Health, Grand-Duchy of Luxembourg

## Abstract

**Background:**

The ORISCAV-LUX study is the first baseline survey of an on-going cardiovascular health monitoring programme in Grand-Duchy of Luxembourg. The main objectives of the present manuscript were 1) to describe the study design and conduct, and 2) to present the salient outcomes of the study, in particular the prevalence of the potentially modifiable and treatable cardiovascular disease risk factors in the adult population residing in Luxembourg.

**Method:**

ORISCAV-LUX is a cross-sectional study based on a random sample of 4496 subjects, stratified by gender, age categories and district, drawn from the national insurance registry of 18-69 years aged Luxembourg residents, assuming a response rate of 30% and a proportion of 5% of institutionalized subjects in each stratum. The cardiovascular health status was assessed by means of a self-administered questionnaire, clinical and anthropometric measures, as well as by blood, urine and hair examinations. The potentially modifiable and treatable risk factors studied included smoking, hypertension, dyslipidemia, diabetes mellitus, and obesity. Both univariate and multivariate statistical analyses used weighted methods to account for the stratified sampling scheme.

**Results:**

A total of 1432 subjects took part in the survey, yielding a participation rate of 32.2%. This figure is higher than the minimal sample size of 1285 subjects as estimated by power calculation. The most predominant cardiovascular risk factors were dyslipidemia (69.9%), hypertension (34.5%), smoking (22.3%), and obesity (20.9%), while diabetes amounted 4.4%. All prevalence rates increased with age (except smoking) with marked gender differences (except diabetes). There was a significant difference in the prevalence of hypertension and of lipid disorders by geographic region of birth. The proportion of subjects cumulating two or more cardiovascular risk factors increased remarkably with age and was more predominant in men than in women (*P*<0.0001). Only 14.7% of men and 23.1% of women were free of any cardiovascular risk factor. High prevalence of non-treated CVRF, notably for hypertension and dyslipidemia, were observed in the study population.

**Conclusion:**

The population-based ORISCAV-LUX survey revealed a high prevalence of potentially modifiable and treatable cardiovascular risk factors among apparently healthy subjects; significant gender and age-specific differences were seen not only for single but also for combined risk factors. From a public health perspective, these preliminary findings stress the urgent need for early routine health examinations, preventive interventions and lifestyle behavioural changes, even in young asymptomatic adults, to decrease cardiovascular morbidity and mortality in Luxembourg.

## Background

Today around 60% of worldwide deaths and 43% of the global burden of diseases are attributed to coronary heart disease, stroke and type 2 diabetes mellitus[1]. These diseases are predicted to account for 73% of global deaths and 60% of the global burden by year 2020[2]. There is strong evidence that cigarette smoking, obesity, lipid disorders, elevated blood pressure and diabetes mellitus are not only associated with each other[3-5] but also with cardiovascular morbidity and mortality[6-14]. On the other hand, it has been shown that major changes in the risk of CVD can be reduced by modifications of the lifestyle and social behaviour of individuals [15,16]. Despite major advances in prevention, diagnosis and treatment measures, cardiovascular disease (CVD) is still the main cause of mortality in Europe. It accounts for over 4 million deaths yearly, i.e., nearly half (49%) of all European deaths, but with striking geographical variations[17].

The Grand-Duchy of Luxembourg is a small country in the heart of Europe landlocked by Belgium, France and Germany, with a population of 493,500 inhabitants (official estimate, 2009) over an area of about 2600 km^2^. Distinctively, Luxembourg country is a multicultural society. Luxembourgish people constitute approximately 56.3% of the population, while 43.7% of the inhabitants are well-integrated foreign residents from over 150 different nationalities, mostly Portuguese (16.2%), French (5.8%), Italians (3.9%) and Belgians (3.4%). Evidently, the national health profile is significantly determined by the health of its immigrants.

In Luxembourg, cardiovascular mortality accounted for about one-third of total causes of death in 2005[18]. According to a recent study comparing the cardiovascular mortality rates in three neighbouring regions, Grand-Duchy of Luxembourg, Wallonia in Belgium, and Lorraine in France, Luxembourg ranked first with elevated cerebro-vascular and ischemic coronary disease mortality in both men and women[19]. This outcome made cardiovascular health as one of the top priorities of Luxembourg healthcare authorities. Although the Ministry of health has long been involved in planning and organizing prevention programs and health promotion campaigns to endorse healthy lifestyles, there has been so far no proper population-based study to assess the prevalence and clustering of cardiovascular risk factors (CVRF) among adults. Luxembourg is one of the few European countries without a well-defined structure for permanent and/or periodic observational surveys on cardiovascular health statistics.

The "Observation of Cardiovascular Risk Factors in Luxembourg" (ORISCAV-LUX) survey, was conducted between November 2007 and January 2009 under the auspices of the Ministry of Health and co-financed by the Ministry of Research. It was designed as a nationwide cross-sectional cardiovascular monitoring survey aimed to establish baseline information on the prevalence of potentially modifiable and preventable CVRF, including obesity, hypertension, diabetes mellitus, lipid disorder, and current smoking, among the general adult population of Luxembourg. From a public health and research perspective, this survey is intended to be repeated at regular intervals to monitor the evolution. Accurate assessment of the distribution of single and combined CVRF not only describes the total burden of CVD but also permits the development of coherent and effective strategies of prevention and treatment.

## Methods

### Sampling scheme

A representative random sample was drawn from the national health insurance registry, stratified by gender (male and female), age (5-year categories) and districts of residence (Luxembourg, Diekirch and Grevenmacher). With a 98% social coverage rate, the registry is considered as the most complete list of inhabitants available in Luxembourg. The minimal necessary representative sample size was calculated to 1285 subjects to ensure statistical power[20], i.e. to ensure a statistical precision of at least 2% for the estimation of the prevalence of the risk factors at the 95% confidence level. However, based on literature review and previous evidence with such multiple-stage population-based studies, a high non-participation rate was expected, including refusal, invalid addresses and non-response. Assuming a response rate of 30% and a proportion of 5% of institutionalized subjects in each stratum, the sample size was augmented to 4496 subjects. The distribution of selected subjects in each stratum was proportional to their distribution in the source population (adult population of 18 to 69 years residents in Luxembourg). Pregnant women, people living in institutions, subjects outside the age range 18-69 years and those deceased before recruitment were excluded. The sample is weighted to account for the complex sampling design and for non-response.

### Method of recruitment

All selected subjects were invited by an official letter signed by healthcare authorities and the research centre for health studies, in which the objectives and the tests to be performed were broadly explained. They were asked to send their primary agreement and phone number by using the prepaid envelope holding the address of the study research centre. The subjects who agreed to take part in the study were contacted by phone to attend the appointment at the nearest medical centre. Two subsequent reminders were sent to those who did not respond spontaneously after 3 weeks. Participants were requested to attend the health examination centre in a fasting state. Home visits were organized in case the participant had health problems or transport difficulties. An informed consent form was handed to the participants at their attendance visit and had to be signed prior to inclusion. The study comprised three major steps: an auto-administered questionnaire, physical and anthropometric measurements, and blood, urine and hair tests. All participants were informed about their measurements and tests results and were advised to consult, their family doctor if necessary. Substantial efforts were made to increase response rate, not only during the survey preparation but also during the recruitment phase. Complete details about non-response management, sample representativeness of the population and comparison of participants and non-participants to ORISCAV-LUX survey are published elsewhere[21]. Briefly, a total of 1432 subjects (32.2%) were successfully recruited, slightly beyond the estimated sample size and the expected response rate. This analysis revealed that the distribution between participants and non-participants was comparable in terms of the cardiovascular morbidity indicators, including prescribed medications, hospital admission and medical measures.

### Questionnaire

The self-administered questionnaire elicited information on demographic and socioeconomic characteristics, including age, gender, country of birth, income, education, profession, and marital status. Data regarding life style characteristics, including cigarette smoking, alcohol consumption, physical activity and dietary habits, as well as family and personal diseases history and medication intake, were also collected. Trained survey nurses helped and checked the answers with the participants. Given the multi-linguistic nature of the population residing in Luxembourg, the self-administered questionnaire was translated from French into the three other most used languages, namely German, English and Portuguese, and then backward translated into French to ensure the validity [22].

### Anthropometric and blood pressure measurements

For blood pressure measurement, subjects were seated in a chair with their arms bared and supported at heart level. Systolic blood pressure (SBP, mmHg) and diastolic blood pressure (DBP, mmHg) were measured at least 3 times with a minimum of 5-min interval between each measurement, by using Omrom^® ^MX3 plus automated oscillometric Blood Pressure Monitor (O-HEM-742-E) (Matsusaka, Japan)[23], with an appropriate cuff size adapted to the upper arm perimeter of participant. Measurements were only performed after the participants had been seated for at least 5 minutes after questionnaire completion and at least 30 minutes after blood intake and refrained from smoking. The average of the last 2 readings was used in the analysis.

Body weight (kg) was recorded with an accuracy of ± 100 g by using a digital column scale (Seca^® ^701, Hamburg, Germany), with subject barefoot and wearing light clothing. The scale was calibrated regularly. Standing body height (cm) was recorded to the nearest 0.2 cm with a portable wall stadiometer (Seca, Germany), attached to the scale, with heels together, shoulders in relaxed position and arms hanging freely. Body mass index (BMI) was calculated as body weight in kg divided by the square of height (m). Self-reported height and weight were not acceptable. Waist circumference (WC, cm) was measured at the level midway between 12th rib and the uppermost lateral border of the iliac crest during mild expiration. Waist circumference was measured to the nearest 0.2 cm with the subject at standing position, using a flexible, non-distensible tape and avoiding pressure exertion on the tissues. The duplicate measurements in a subgroup of participants showed high reproducibility (data not shown).

### Biochemical parameters

A venous blood sample was drawn from the arm of each subject in sitting position by antecubital vein puncture, after an overnight 8-hour fast. The blood samples were transferred to the core Laboratory of the "Centre Hospitalier in Luxembourg" (CHL) in tank containing ice packs to maintain a suitable temperature. They were centrifuged within maximum 4 hours after extraction and then immediately analysed. Blood collection tubes containing glycolytic inhibitors were used for serum glucose test. The Laboratory applies strict internal and external standard quality control techniques. The laboratory tests performed included fasting plasma glucose (FPG, mg/dl), triglycerides (TG, mg/dl)), total cholesterol (TC, mg/dl), low-density lipoprotein cholesterol (LDL-C, mg/dl), high-density lipoprotein Cholesterol (HDL-C, mg/dl) and other biomarkers.

### CVD risk factors

Based on the International Obesity Task Force[24], convened by the World Health Organization, a subject with BMI ≥ 30.0 kg/m^2 ^was defined as obese. The WC measurements were used to determine the extent of central adiposity, with cut-off points of ≥ 102 cm in men and ≥ 88 cm in women[25]. Participants were classified as having elevated blood pressure if they reported taking anti-hypertensive medications and/or had SBP ≥ 140 mmHg and/or DBP ≥ 90 mmHg [26].Participants were classified as diabetics if they reported taking anti-diabetic medications and/or had FPG ≥ 126 mg/dl [27,28]. Subjects with lipid disorder were defined as having at least one of the following anomalies: TC ≥ 190 mg/dl, TG ≥ 150 mg/dl, LDL-C ≥ 115 mg/dl, and HDL-C < 40 mg/dl for men and < 46 mg/dl for women [29], and/or taking hypo-lipid medications. Smoking status was evaluated by the self-reported questionnaire. Each participant was classified as current smoker, ex-smoker or non-smoker. Current regular smokers were those who smoked at least one cigarette per day. Current occasional smoker were those who smoked less than one cigarette per day. The present study specifically focused on 5 distinct CVRF, respectively, obesity, hypertension, diabetes mellitus, lipid disorder, and current smoking. The metabolic syndrome (MS) was succinctly presented according to the simplest and most used R-ATP III definition[30]; a participants is defined as having the MS when three or more of the following criteria are met: 1) WC ≥ 102 cm for men and ≥ 88 cm for women; 2) raised concentration of TG ≥ 150 mg/dl or specific treatment for this lipid anomaly; 3) reduced concentration of HDL-C < 40 mg/dl for men and < 50 mg/dl for women or specific treatment for this lipid anomaly; 4) SBP was ≥ 130 mmHg, or DBP ≥ 85 mmHg or treatment of previously diagnosed hypertension; 5) FPG level ≥100 mg/dl or use of medication for hyperglycemia.

### Quality control procedure

Strict control measures were applied to ensure quality throughout the conduct of the study, namely, sample selection, operational data-collection, data processing and reporting. They included well-defined sampling design and prior estimation of sample size to reduce sampling errors; testing and validating the translated questionnaires; introducing multiple cross-checked questions on the same topic to validate the results; training of data collection nurses to perform their duties according to standard operating procedures; monitoring and ascertaining of instruments performance every morning before starting the tests on the subjects; daily assessment of paper forms for answer's relevance and completeness; designing the database in a way that allows to confirm the validity of the participant's identification codes, establishing the completeness of the entered data and performing basic data checks; training of data processing personnel to be able to provide reliable and accurate documentation, as well as periodic summary reports on the latest project progress; independent double data entry followed by matching and checking for data-entry errors, so that problems were appropriately remedied; data cleaning according to experts consensus; finally, checking of the internal and external consistency of the analyzed data before reporting.

### Ethical aspects

All participants were duly informed and consented to take part in the study. The study design and information collected were approved by the National Research Ethics Committee and the National Commission for Private Data Protection.

### Statistical analysis

Results were expressed as mean ± SE for quantitative variables and as count and proportion (%) for categorical variables. Multiple regression analysis was used to test the effect of age, gender and their interaction on subject's quantitative characteristics. Instead, when considering CVRF binary outcomes, logistic regression was applied. Given that the present study focused specifically on 5 CVRF (obesity, hypertension, diabetes mellitus, lipid disorder, and current smoking), the number of CVRF was calculated for each subject and analysed as a discrete variable ranging from 0 (no CVRF) to 5 (all CVRF present). To account for the stratified random sampling method used to recruit the subjects, weighted statistical methods were applied. A sampling weight equal to the inverse probability of unit selection was allocated to each subject from the same stratum. This stratum sampling weight was defined as the ratio between the population stratum size and the observed sample stratum size. Results were considered to be significant at the 5% critical level (*P < 0.05*). All statistical analyses were performed by using the SAS 9.2 (^© ^SAS Institute Inc., Cary, NC, USA).

## Results

Among the random sample of 4496 subjects aged 18-69 years drawn from Luxembourg residents population, 1432 (697 men and 735 women) responded and were included in the study. All participants filled out the questionnaire; however, blood pressure readings were missing for 2 subjects and anthropometric measurements for one subject. Blood, urine and hair samples were obtained from, respectively, 1427 (99.7%), 1432 (100%) and 1062 (74.1%) participants.

The anthropometric, clinical and biochemical characteristics of these subjects are displayed in Table 1 by age category and gender. As expected, mean values of BMI, WC, SBP, DBP, FPG, TC, LDL-C, and TG increased significantly with age; HDL-C levels, however, remained quite stable across age categories (*P *= 0.64). A significant gender-specific difference was observed for BMI, WC, SBP, FPG, HDL-C, LDL-C and TG, systematically higher in men than in women (*P *< 0.0001), except for TC (*P *= 0.94). For DBP, a significant age-gender interaction was found (*P *= 0.0005), in the sense that DBP increased more markedly with age in men than in women.

**Table 1:** Anthropometric, clinical and biochemical characteristics by age category and gender in the ORISCAV-LUX study

Characteristic		18-29 y	30-39 y	40-49 y	50-59 y	60-69 y	*P*-value*	*P*-value**
Number of subjects (men/women)		221 (106/115)	351 (171/180)	372 (189/183)	284 (135/149)	204 (96/108)		

BMI (kg/m^2^)	Men	24.1 ± 0.3	26.6 ± 0.3	27.7 ± 0.3	28.6 ± 0.4	29.1 ± 0.5	<0.0001	<0.0001
	Women	22.9 ± 0.4	25.3 ± 0.3	25.9 ± 0.4	26.6 ± 0.4	29.1 ± 0.6		
								
WC (cm)	Men	83.5 ± 1.0	91.6 ± 0.8	96.7 ± 1.0	99.4 ± 1.0	102.0 ± 1.3	<0.0001	<0.0001
	Women	76.6 ± 1.0	83.1 ± 0.9	84.3 ± 0.9	87.7 ± 1.0	93.8 ± 1.4		
								
SBP (mmHg)	Men	124.3 ± 1.1	127.5 ± 1.0	134.0 ± 1.1	139.5 ± 1.3	149.7 ± 1.9	<0.0001	<0.0001
	Women	114.8 ± 0.8	116.9 ± 0.9	124.0 ± 1.1	131.8 ± 1.3	142.8 ± 2.0		
								
DBP (mmHg)	Men	74.1 ± 0.7	81.9 ± 0.8	87.1 ± 0.8	88.2 ± 0.8	88.5 ± 1.2	<0.0001^$^	0.26^$^
	Women	74.8 ± 0.8	77.1 ± 0.8	81.2 ± 0.8	83.7 ± 0.8	84.3 ± 1.1		
								
FPG (mg/dl)	Men	89.6 ± 1.0	91.9 ±0.7	97.7 ± 0.9	106.2 ± 2.3	108.5 ± 2.7	<0.0001	<0.0001
	Women	84.4 ± 0.6	86.8 ± 0.6	92.2 ± 0.9	96.3 ± 1.8	99.9 ± 2.4		
								
TC (mg/dl)	Men	170.5 ± 3.4	198.3 ± 2.9	214.5 ± 2.9	210.1 ± 3.2	198.9 ± 4.2	<0.0001	0.94
	Women	177.7 ± 3.3	189.9 ± 2.9	199.1 ± 2.5	220.3 ±2.7	217.1 ± 4.5		
								
HDL-C (mg/dl)	Men	53.9 ± 1.2	53.2 ± 0.9	53.1 ± 1.1	53.9 ± 1.2	54.7 ± 1.5	0.64	<0.0001
	Women	68.4 ± 1.4	67.9 ± 1.2	67.6 ± 1.2	69.1 ± 1.5	69.4 ± 2.0		
								
LDL-C (mg/dl)	Men	104.1 ± 2.9	126.1 ± 2.3	140.1 ± 2.6	137.0 ± 2.8	127.8 ± 3.9	<0.0001	<0.0001
	Women	98.2 ± 2.8	109.9 ± 2.3	119.5 ± 2.2	135.5 ± 2.5	132.4 ± 4.0		
								
TG (mg/dl)	Men	95.4 ± 6.5	138.9 ± 11.2	149.3 ± 7.5	140.5 ± 6.5	134.4 ± 6.0	<0.0001	<0.0001
	Women	85.8 ± 3.8	97.8 ± 11.5	89.6 ± 3.6	105.7 ± 5.1	107.7 ± 5.6		

Results are expressed as Mean ± SE. * *P*-value for age, ** *P*-value for gender, $ adjusted for age and sex interaction when necessary

Table 2 displays the overall, age-and gender-specific prevalences of each of the five CVRF on which the study focused in addition to the MS. By decreasing prevalence, the most predominant cardiovascular risk factors were lipid disorder (69.9%), hypertension (34.5%), current smoking (22.3%), obesity (20.9%), and diabetes (4.4%), respectively. The prevalence of hypertension, obesity, diabetes mellitus and lipid disorders increased remarkably with advancing age (*P*<0.0001). By contrast, the prevalence of current smoking decreased significantly with age (*P*<0.0001). Further, the prevalence was significantly higher in men than in women for hypertension (41.9% vs 27.1%), obesity (23.0% vs 18.7%), lipid disorders (74.3% vs 65.5%), and current smoking (24.9% vs 19.7%), as well so for MS (29.5% vs 18.4%), but not for diabetes (5.2% vs 3.5%, *P*= 0.08) (data not shown). No age-gender interaction was found for any of the CVRF. While the prevalence of hypertension and of lipid disorders differed significantly by geographic region of birth, no significant difference was observed for diabetes, obesity and current smoking (Table 3).

**Table 2:** Prevalence of cardiovascular risk factors (CVRF) by age category and gender in the ORISCAV-LUX study

CVRF		18-69 n (%)	18-29 n (%)	30-39 n (%)	40-49 n (%)	50-59 n (%)	60-69 n (%)	*P*-value*	*P*-value**
Obesity	Total	325 (20.9)							
	Men	175 (23)	7 (6.3)	35 (20.3)	50 (26.6)	45 (32.9)	38 38.6)	<0.0001	0.03
	Women	150 (18.7)	7 (6.7)	28 14.2)	40 (20.6)	32 (22.2)	43 (39.4)		
									
Hypertension	Total	540 (34.5)							
	Men	322 (41.9)	10 (9.3)	50 (28.7)	95 (50.2)	86 (63.0)	81 (84.1)	<0.0001	<0.0001
	Women	218 (27.1)	7 (6.3)	22 (13.1)	51 (26.7)	64 (43.5)	74 (67.8)		
									
Diabetes	Total	69 (4.4)							
	Men	39 (5.2)	1 (1.1)	1 (0.7)	7 (3.8)	12 (8.8)	18 (18.8)	<0.0001	0.08
	Women	30 (3.5%)	0 (-)	1 (0.5)	5 (2.4)	8 (5.3)	16 (14.7)		
									
Lipid disorder	Total	1033 (69.9)							
	Men	533 (74.3)	44 (42.7)	119 (70.9)	159 (84.5)	120 (89.7)	91 (96.5)	<0.0001	0.0002
	Women	500 (65.5)	48 (42.3)	93 (52.0)	124 (67.8)	136 (92.0)	99 (92.0)		
									
Current smoking	Total	307 (22.3)							
	Men	165 (24.9)	41 (38.6)	39 (22.7)	46 (24.5)	30 (22.4)	9 (8.6)	<0.0001	0.03
	Women	142 (19.7)	33 (28.7)	32 (17.3)	31 (16.7)	31 (20.3)	15 (13.0)		
									
R-ATPIII-defined MS	Total	368 (24.0)	9 (4.4)	40 (11.6)	95 (25.5)	114 (42.1)	110 (55.1)	<0.0001	<0.0001
	Men	221 (29.5)	7 (6.9)	26 (15.6)	62 (33.8)	67 (50.5)	59 (61.2)		
	Women	147 (18.4)	2 (2.0)	14 (7.5)	33 (17.1)	47 (33.3)	51 (49.4)		

Results are expressed as number of cases (%). * P value for age, ** P-value for gender

**Table 3:** Prevalence of CVRF by geographic region of birth adjusted for age, gender and district

CVRF	Luxembourg (n = 869)	Portugal (n = 169)	Europe (n = 311)	Others (n = 83)	P-value
Obesity	21.5% ± 1.3	23.6% ± 3.2	18.4% ± 2.2	17.7% ± 4.2	0.45
Hypertension	37.1% ± 1.5	37.6% ± 3.7	28.1% ± 2.5	25.4% ± 4.7	0.009
Diabetes	4.7% ± 0.7	3.1% ± 1.3	4.7% ± 1.1	2.1% ± 1.4	0.51
Lipid disorder	70.5% ± 1.6	68.3% ± 3.8	73.3% ± 5.8	56.3% ± 2.6	0.039
Current smoking	23.1% ± 1.5	21.4% ± 3.4	21.1% ± 2.4	20.4% ± 4.7	0.86

Results are expressed as percents ± SE

**Figure 1:**
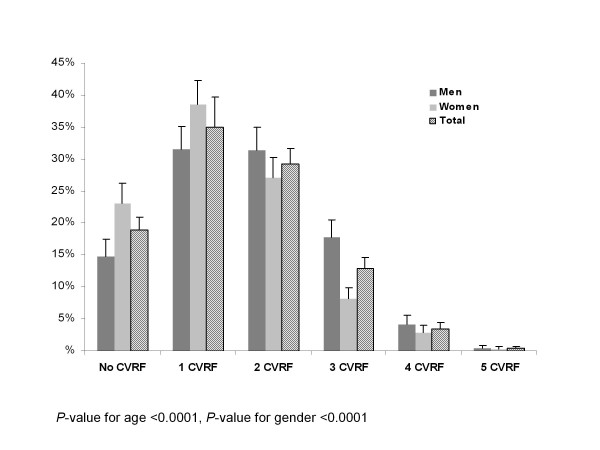
**Distribution of the number of CVRF per subject globally and by gender in the ORISCAV-LUX study**.

The distribution of the number of CVRF affecting each subject is displayed in Figure 1, globally and by gender. It is seen that 35% of the participants presented only one CVRF, 29.3% presented 2 CVRF, and 16.9% presented 3 and more CVRF. Only 18.9% of the subjects (14.7% of men and 23.1% of women) were free of any CVRF. Among subjects presenting only one CVRF, 75% had lipid disorder, 11.4% were regular smokers and 9.5% suffered hypertension. For individuals with 2 CVRF, the most frequent combinations were lipid disorder and hypertension (53%), lipid disorder and smoking (24.2%), lipid disorder and obesity (14.4%). In subjects with 3 CVRF, the "lipid disorder, hypertension and obesity" combination was found in 64.9% of the cases, while the "lipid disorder, hypertension and current smoking" profile was observed in 13.9% of the subjects. Finally, in subjects with 4 CVRF, it was found that 55.6% of them were exempt of current smoking and 38.9% were exempt of diabetes. When analysing the number of CVRF per participant with respect to age and gender, it was observed that this number increased significantly with age (*P* < 0.0001), and was more predominant in men than in women (*P* < 0.0001). The interaction term between age and gender was not significant.

**Figure 2:**
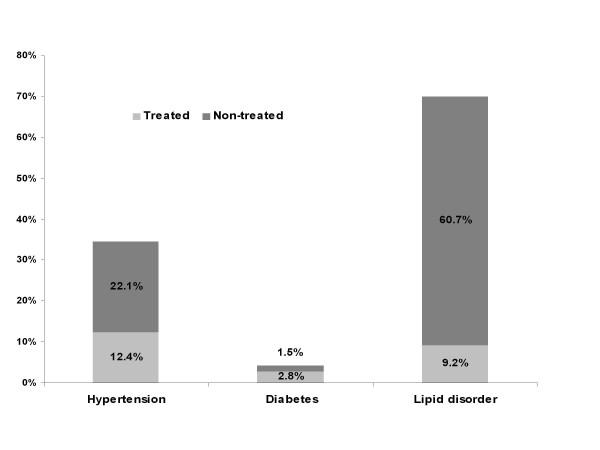
**Prevalence of treated and non-treated participants with hypertension, diabetes and lipid disorder in the ORISCAV-LUX study**.

Figure 2 illustrates the prevalence of treated and non-treated participants with hypertension, diabetes and lipid disorder. The prevalence of hypertension among the resident population was 34.5%; about two thirds of the participants (22.1%) were not treated. Among the 4.4% participants having the diabetes, 2.8% were treated against 1.5% non-treated. Among those with lipid disorder (69.9%), only 9.2% were under medication.

## Discussion

In Grand-Duchy of Luxembourg, CVD is the primary cause of mortality and a major reason for hospital admission. This has led public health authorities to consider CVD as one of their national healthcare priorities. It is well established that early management of CVRF by lifestyle modifications and/or therapeutic interventions leads to marked reduction in mortality, morbidity, and maintaining quality of life [31,32]. Therefore, early detection of individuals with modifiable and treatable CVRF may result in saving lives and reducing the burden on healthcare resources. In the absence of relevant national baseline statistics, the observation of CVRF by national periodic surveys is an essential tool to formulate coherent and effective strategies of prevention.

This paper describes the rationale, the objectives, the method of recruitment and the primary outcomes of the ORISCAV-LUX study, the first national large-scale epidemiological survey with a representative sample of 18-69 years old adults residing in Luxembourg. Our findings indicate that several CVRF are highly prevalent conditions, specifically, lipid disorder, followed by hypertension, smoking, obesity, and diabetes. The great variations in published data make direct international comparisons exceedingly difficult, not only because of important methodological differences with respect to the characteristics of the target population, the study design, the sample selection, and the year of conduct, but also due to the multifactorial origin of this health problem and the particularity of health systems. Globally, high CVRF prevalence rates were reported in industrialized countries. According to the most recent statistics from Canada, 26% of Canadians were current smokers, 14.9% were obese, 13.0% had hypertension and 4.2% had diabetes [33].

The prevalence was significantly higher in men than in women for all conditions, except for diabetes. Interestingly, the gender-specific prevalence rates found in the ORISCAV-LUX study were consistent with previously reported findings in European countries, such as Italy[34], Spain[35], Switzerland [36] and Sweden[37].

According to ORISCAV-LUX findings, lipid disorder was the most prominent risk factor for both genders and in all age categories. This observation should nevertheless be interpreted with precaution, in particular by bearing in mind the very strict thresholds regarding the prophylactic medication of subjects who would otherwise be considered formerly at lower risk. Although the variety of definitions complicates comparison with previous studies of adult populations, these percentages are among the highest reported in the literature and might also explain the higher prevalence of obesity. The high prevalence of dyslipidemia calls for an early screening of lipid disorders and for an evaluation of current national nutritional recommendations.

In the present study, there was no significant difference in the prevalence of diabetes between men and women. However, the frequency of the disease increased with age. Although this finding was consistent with that of Switzerland study [36], the overall recorded diabetes rate was lower in Luxembourg (4.4% against 6.6%, respectively).

In USA, smoking is the leading preventable cause of death[38]. Despite the difficulty to compare tobacco consumption on international level, the overall age-adjusted prevalence of smoking in 21 European countries was 33.1% in men and 29% in women, with major variations between country samples[39]. In the ORISCAV-LUX survey, about a quarter of the participants were current (occasional and regular) smokers. Globally, the prevalence of smoking was significantly higher in men than in women and decreased with age. Interestingly, these findings were comparable to those of the national tobacco consumption survey, carried out annually by the national foundation of fight against cancer. The increasing awareness of the damaging health effects of smoking, together with the anti-smoking measures of the government, including intensified anti-smoking campaigns, the banning of advertising and taxation, contributed widely to the declining trend of smoking among adults over the last two decades. From a public health point of view, it is important to maintain this progression since 54% of world ischemic heart diseases and 25% of world cardiovascular mortality cases were attributed to smoking[40].

Luxembourg is characterized by its multicultural nature. Among Europeans, the Portuguese represent the largest segment of the population. The preliminary ORISCAV-LUX findings showed that the prevalence of two CVRF studied, specifically the hypertension and lipid disorders, differed significantly between countries of birth (Luxembourg, Portugal, Europe and others). Characterising groups at risk in the general population is important to enable tailoring public health action accordingly. However, this issue needs further research to support the hypothesis that the risk of CVD is influenced by the country of birth and that immigrants tend to have better health[41].

It has been recognized that CVRF are associated with each other and their effects tend to be synergistic rather than additive[5,42,43]. Thus, the assessment of multiple risk factors is crucial to identify a high risk population. The metabolic syndrome is the most suggested approach to study the clustering of CVRF in a population. The overall prevalence of MS, estimated according to R-ATP III criteria, was 24% among the participants aged 18-69 years residing in Luxembourg. In scope of the present manuscript, the focus was primarily on the number of CVRF per individual. Further data on the metabolic syndrome will be published separately. Interestingly, it was found that combinations of two or more CVRF were more frequently observed in men than in women and increased remarkably with age. Moreover, multiple risk profiles already emerged within the young adults aged 18-29 years (data not shown). These findings suggest that cumulated CVRF is quite common in the Luxembourg's apparently healthy adult population and appears to be already present in young men and to persist thereafter. These observations were consistent with results from previous recent studies[44,36,45]. High prevalence of non-treated CVRF, notably of the hypertension and dyslipidemia were observed in the study population. Although further in-depth analysis is necessary, these preliminary findings underline the necessity to increase disease awareness, to improve screening of high risk subjects and to promote prevention both at the public and individual levels.

The strengths of the ORISCAV-LUX study reside firstly in the population sample; the small size of the country made it possible to organize data collection at a national level. Secondly, a complementary study comparing the known demographic and cardiovascular health-related profiles of participants and non-participants demonstrated that the participants did not differ substantially from non participants. Given the absence of discriminating health profiles between participants and non-participants, it was concluded that the response rate did not invalidate the results and allowed generalizing the findings for the entire population (detailed results on this comparison are published separately). Thirdly, the conception of the survey was consistent with the WHO stepwise approach[46], which recommends measuring chronic diseases risk factors by using standardized tools and objective measurements to facilitate national and international comparability. To avoid inaccurate retrieving of CVRF from self-administered information, this approach permits to provide reliable objective estimates of the prevalence of major CVRF, namely, obesity, diabetes, hypertension and lipid disorders.

### Perspectives

Further analyses of the ORISCAV-LUX database are currently ongoing. In particular, the assessment of the prevalence of the metabolic syndrome and its associated socioeconomic determinants, as well as its interaction with dietary habits, alcohol consumption and physical activity are important topics to be studied in more detail. A continuous monitoring of the CVRF with clearly specified research and public health objectives is planned to study their trends over time.

### Conclusion

According to the current guidelines, the ORISCAV-LUX survey documents for the first time a remarkably high prevalence of major CVRF among the adult residents in Luxembourg. In addition, the survey data confirm significant age-gender differences as well as individual CVRF profiles. The presence of 2 or more CVRF per subject was more predominant in men than in women, while the younger age group (18-29 years) already presented a striking multiple risk profile.

The ORISCAV-LUX survey constituted a timely opportunity to identify public health determinants, hence to help shaping policies according to preventive needs of the population. Its first outcomes underscore the need to identify other characteristics of high-risk population, to continue epidemiological monitoring and to implement population-based multiple-facet interventions tailored for special groups at risk.

## Abbreviations

ORISCAV-LUX: Observation of Cardiovascular Risk Factors in Luxembourg; CVD: cardiovascular disease; CVRF: cardiovascular risk factors; BP: Blood pressure; SBP: systolic blood pressure; DBP: Diastolic blood pressure; BMI: Body masse index; LDL-C: Low-density lipoprotein cholesterol; HDL-C: High-density lipoprotein cholesterol; TG: Triglycerides; FPG: Fasting Plasma Glucose; WC: Waist circumference; BMI: Body Mass Index; Metabolic syndrome: MS; R-ATPIII: Revised National Cholesterol Education Programme-Adult Treatment Panel III.

## Competing interests

The authors declare that they have no competing interests.

## Authors' contributions

AA involved in the conception and design of the ORISCAV-LUX survey, coordinated the field data collection, data analyses and drafted the manuscript. NS conducted the statistical analyses. A-FD participated in the statistical analyses and critical discussion of the results. SC involved in the critical discussion of results. JB and CD are cardiologists and medical investigators involved in the discussion of results. YW involved in the critical discussion of results. M-LL involved in the conception, design of the ORISCAV-LUX survey, and in the critical discussion of results. AAlbert contributed to the repeated critical revision of the manuscript and intellectual content. MG provided expertise and oversight throughout the process. All of the authors reviewed drafts and approved the final version of the manuscript.

## Pre-publication history

The pre-publication history for this paper can be accessed here:

http://www.biomedcentral.com/1471-2458/10/468/prepub
